# Collagen hydrogels loaded with fibroblast growth factor-2 as a bridge to repair brain vessels in organotypic brain slices

**DOI:** 10.1007/s00221-020-05907-7

**Published:** 2020-08-29

**Authors:** Buket Ucar, Sedef Yusufogullari, Christian Humpel

**Affiliations:** 1 Laboratory of Psychiatry and Experimental Alzheimer’s Research, Department of Psychiatry and Psychotherapy, Medical University of Innsbruck, Anichstrasse 35, 6020 Innsbruck, Austria; 2 Biomedical Institute, Gebze Technical University, Istanbul, Turkey

**Keywords:** Brain vessel, Repair, Collagen hydrogel, Organotypic brain slices, Fibroblast growth factor-2

## Abstract

Vessel damage is a general pathological process in many neurodegenerative disorders, as well as spinal cord injury, stroke, or trauma. Biomaterials can present novel tools to repair and regenerate damaged vessels. The aim of the present study is to test collagen hydrogels loaded with different angiogenic factors to study vessel repair in organotypic brain slice cultures. In the experimental set up I, we made a cut on the organotypic brain slice and tested re-growth of laminin + vessels. In the experimental set up II, we cultured two half brain slices with a gap with a collagen hydrogel placed in between to study endothelial cell migration. In the experimental set up I, we showed that the number of vessels crossing the cut was tendencially increased with the addition of fibroblast growth factor-2 (FGF-2), vascular endothelial growth factor, or platelet-derived growth factor-BB compared to the control group. In the experimental set up II, we demonstrated that a collagen hydrogel loaded with FGF-2 resulted in a significantly increased number of migrated laminin + cells in the gap between the slices compared to the control hydrogel. Co-administration of several growth factors did not further potentiate the effects. Taken together, we show that organotypic brain slices are good models to study brain vessels and FGF-2 is a potent angiogenic factor for endothelial cell proliferation and migration. Our results provide evidence that the collagen hydrogels can be used as an extracellular matrix for the vascular endothelial cells.

## Introduction

The human brain contains approximately 650 km-long capillaries, so much that it is estimated that each neuron is perfused by its own capillary (Zlokovic 2005; Cipolla 2009). The brain capillary network includes primarily endothelial cells and pericytes, which are surrounded by astrocytic end-feet, forming a neurovascular unit (Cipolla 2009). Endothelial cells line all the vessels from big arteries and veins to the smallest capillaries in the whole body, regulating the diffusion of molecules in circulation. Brain endothelial cells form the blood–brain barrier (BBB) and tightly regulate the transport between the brain and the periphery. Proper functioning of neurons depends on this tight regulation of the extracellular environment and impairments of BBB disrupts central nervous system (CNS) homeostasis. Vascular damage in the brain is associated with various pathologies, including neurodegenerative diseases, microbleeds, ischemic stroke, cognitive problems, and BBB disorders (Hu et al. 2017). In spinal cord injury, rupture of the blood vessels causes hemorrhages and loss of tissue (Oudega 2012).

Endothelial cells under normal circumstances proliferate very slowly, for a mouse brain once a couple of years (Alberts et al. 2002). However, in case of injury or other external stimuli such as angiogenic factors and inflammatory molecules, proliferation of endothelial cells accelerates. After sustaining a vessel injury, angiogenic mechanisms take action which, however, mostly fail to restore the previous function. To study endothelial cell proliferation and migration, aortic ring models are one of the most common methods, in which a ring of the aorta is embedded in extracellular matrix (Bellacen and Lewis 2009). Matrigel, a reconstituted basement membrane preparation, is one of the most frequently used extracellular matrices for 2D and 3D in vitro cultures, besides some in vivo applications. However, Matrigel is not a well-defined matrix with variable amounts of growth factors from batch to batch, as well as the presence of proteins with unknown functions, which may cause variability among experiments (Vukicevic et al. 1992; Hughes et al. 2010). Therefore, it is beneficial to come up with alternative in vitro extracellular matrices with a precisely defined composition to clearly identify the effects of experimental conditions without interference of matrix variability. Type I collagen was used as an extracellular matrix in ex vivo models of aortic ring organ cultures for evaluating endothelial cell sprouting and angiogenesis (Kapoor et al. 2020). Collagen is a natural extracellular matrix protein with remarkable biocompatibility and no toxicity (for a review see: Ucar and Humpel 2018). We have previously used biodegradable collagen hydrogels crosslinked with polyethyleneglycol (PEG) for controlled and longer term release of other growth factors with the aim of showing neuroprotection, namely nerve growth factor (NGF) for cholinergic neurons and glial cell line-derived neurotrophic factor (GDNF) for dopaminergic neurons (Foidl et al. 2018; Ucar and Humpel 2019). Collagen hydrogels can provide a precisely defined and cost-effective extracellular matrix for endothelial cell growth, as well as providing delivery of angiogenic factors such as fibroblast growth factor-2 (FGF-2).

FGF-2 (or basic FGF) is involved in various cellular functions regulating proliferation, differentiation, survival, and migration (Montesano et al. 1986; Slavin 1995). It is a strong mitogen and chemoattractant for both endothelial cells and smooth muscle cells in vasculature (Slavin 1995). FGF-2 also stimulates pericyte proliferation and production of proteinases from endothelial cells, which can locally degrade the extracellular matrix, allowing cells to migrate for the formation of the new vessels (Slavin 1995; Presta et al. 2005). FGF-2 was recognized for a long time as a potent angiogenic factor supported by the results showing that it induced formation of tubular structures in endothelial cell cultures in vitro and various angiogenic responses and vascular regeneration in vivo (Montesano et al. 1986; Park and Hollenberg 1989; Khurana and Simons 2003). Vascular endothelial growth factor (VEGF) is the best characterized and most studied angiogenic factor due to its role in tumor growth. VEGF was shown to be a potent angiogenic factor by promoting the survival and growth of vascular endothelial cells (Ferrara et al. 2003). The platelet-derived growth factor family (PDGFs) comprises homodimers or heterodimers of four polypeptides: PDGF-A/B/C/D. Among these, PDGF-BB induces the recruitment of smooth muscle cells and pericytes to the vessels (D’Amore and Smith 1993) and modulates endothelial cell proliferation (Battegay et al. 1994). FGF-2, VEGF, and PDGF-BB demonstrated angiogenic activities and vessel repair both for blood and lymphatic vessels (Cao et al. 2004). These factors were also studied in combinations in various models and synergistic effects of FGF-2 and VEGF, as well as FGF-2 and PDGF-BB are well documented (Pepper et al. 1992, 1998; Asahara et al. 1995; Cao et al. 2003).

Organotypic brain slices constitute a link between in vitro and in vivo models, in that they preserve the complex structural interconnection of the brain tissue while allowing direct manipulation and observation (for a review, see: Humpel 2015). Organotypic brain slices have been shown to maintain the complex vasculature of the brain, even in the absence of blood flow (Moser et al. 2003). The cerebral vessels in organotypic brain slices can be successfully stained for basement membrane markers laminin, collagen IV, isolectin IB4, pericyte marker PDFGRβ, smooth muscle and pericyte marker α-smooth muscle actin (αSMA), as well as BBB markers claudin and occludin (Moser et al. 2003; Bendfeldt et al. 2007; Hutter-Schmidt et al. 2015). Thus, organotypic brain slices are a suitable tool to investigate vascularization in the brain. In this current study, we explore the potential use of collagen hydrogels as an extracellular matrix environment for endothelial cell proliferation, migration, and possible new vessel formation in organotypic brain slices by loading the hydrogels with angiogenic factors FGF-2, VEGF, and PDGF-BB.

## Materials and methods

### Organotypic brain slice cultures

**Fig. 1:**
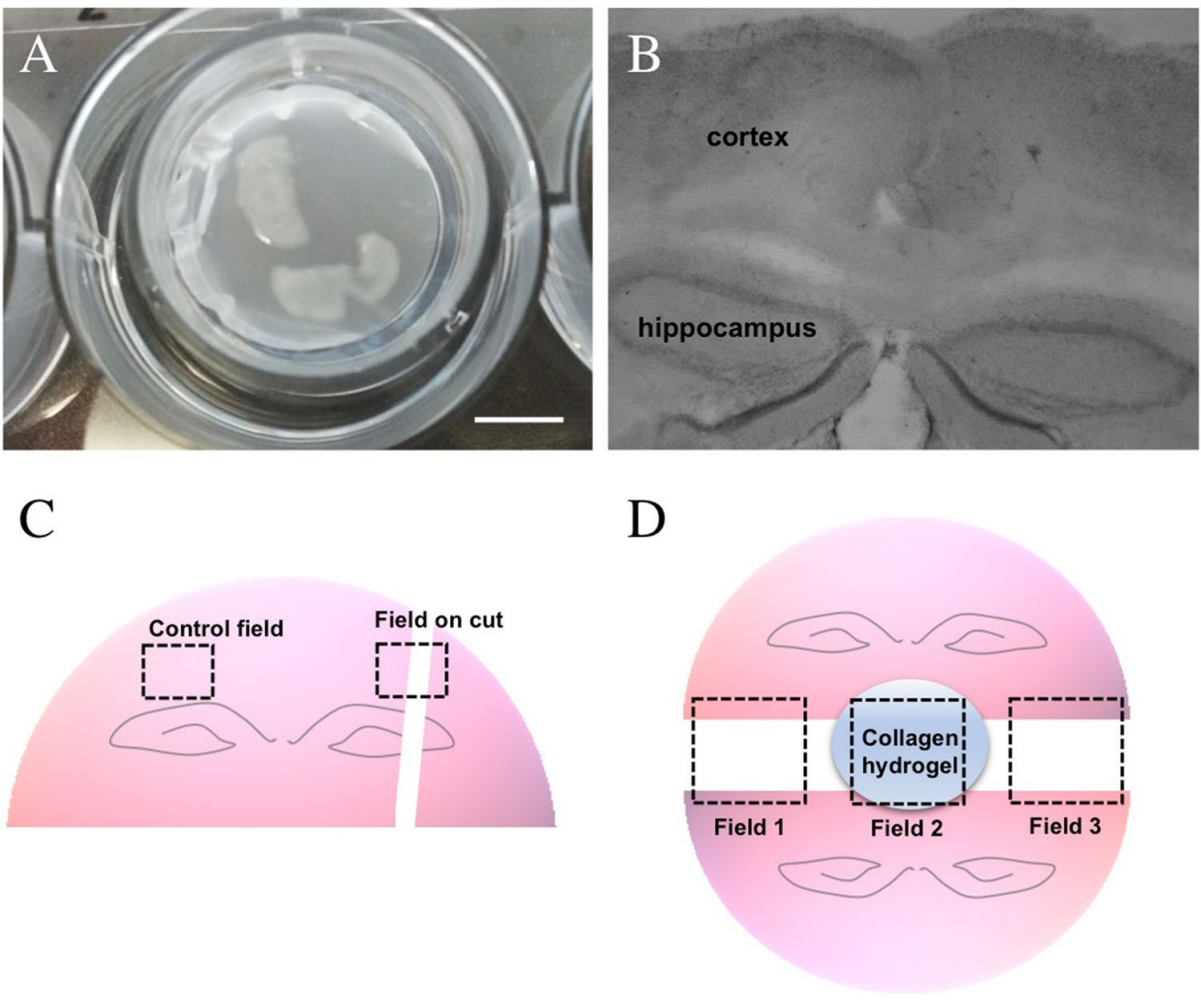
Organotypic half brain slices of 10 day old postnatal mice were prepared at the hippocampal level and cultured on semipermeable membrane inserts (**a**). After 2 weeks in culture, some slices were fixed and stained with cresyl violet (**b**). In experimental set I, a scapel cut was made on the right side and the vessel density was quantified on two fields (**c**). In experimental set II, two slices were placed onto the membrane with a distance of 1.5 mm and a collagen hydrogel was applied between the slices (**d**). Scale bar in **a**: 7.5 mm (**a**) and 514 µm (**b**)

Organotypic brain slices were prepared as described in detail previously by us (Foidl et al. 2018). Briefly, 9–11 day old postnatal C57BL/6 mouse pups were rapidly decapitated and brains were dissected. Brains were glued on the platform of a water-cooled vibratome (Leica VT1000A) and 150 μm-thick slices were cut at the hippocampal level. Half brain vibrosections were placed on membrane inserts (Millipore PICM03050), and cultured in 6-well plates (Greiner) (Fig. 1a). Optionally, slices were placed onto additional membranes (Merck, HTTP02500). Each well contained 1 ml of culture medium that contains 50% MEM/HEPES (Gibco), 25% heat-inactivated horse serum (Gibco/Lifetech), 25% Hanks’ solution (Gibco), 2 mM NaHCO_3_ (Merck), 6.5 mg/ml glucose (Merck), and 2 mM glutamine (Merck), pH 7.2. Brain slices were incubated at 37 °C, 5% CO_2_ for 2 or 8 weeks and culture media were changed once a week. Slices were incubated in culture media without any supplement (control) or with 100 ng/ml murine VEGF (Peprotech, #450-32-2ug), with murine FGF-2 (bFGF, Peprotech, #450-33-10ug) or with murine PDGF-BB (Peprotech, #315-18-2ug). Slices with apparent hippocampi were used for all experiments (Fig. 1b). In the experimental set I, a cut was made on the left side of the brain slice (Fig. 1c). In the experimental set II, 2 half brain slices were placed on the membrane with a distance of approximately 1.5 mm, and collagen hydrogels were placed in the middle of the gap between the slices (Fig. 1d). Our study using animals (mice) follows ethical guidelines for killing animals and our animal work is in compliance with international and national regulations. All work was done to follow the 3Rs (reduce-refine-replace) rules of animal experiments.

### Collagen hydrogels

Collagen hydrogels were prepared as described by us (Foidl et al. 2018). Briefly, a hydrogel solution was prepared with 2 mg/ml bovine collagen type I (Collagen Solutions, UK), and 0.4 mM poly(ethylene glycol) succinimidyl succinate (4S-StarPEG) (Sigma, JKA7006-1G) as a crosslinker in phosphate-buffered saline (PBS). Recombinant murine FGF-2, VEGF, or PDGF-BB were added to this collagen-PEG-PBS solution to the final concentration of 5 ng/μl in solutions for respectively loaded hydrogels and the same amount of PBS was added for generating control hydrogels. During the preparation of the collagen hydrogels, all components were kept on ice all the time to prevent premature gel formation. The pH of the solution was set to 7.2 and 2 μl of droplets from the hydrogel solution were pipetted onto previously sterilized teflon tapes. Droplets of collagen hydrogels were incubated at 37 °C for 1 h for gel formation. Collagen hydrogels that were either empty (control) or containing 10 ng angiogenic factor per 2 µl hydrogel were directly placed onto the membrane in the gap between the two coronal brain slices. For further experiments, collagen hydrogels were prepared with the combination of angiogenic factors by loading 10 ng/scaffold for each of the two or three angiogenic factors.

### Immunohistochemistry

Immunohistochemistry of the brain slices was performed as previously described (Foidl et al. 2018). At the end of a 2 or 8 week culturing period, the organotypic brain slices were fixed with 4% paraformaldehyde for 3 h and washed 3 × with 10 mM PBS. Slices were incubated for 30 min in 0.1% Triton-PBS (T-PBS) at room temperature (RT) and washed 3 × with PBS. Slices were blocked in 20% horse serum/0.2% bovine serum albumin (BSA)/T-PBS for 30 min at RT and subsequently incubated with primary antibody against laminin (1:500, Sigma, L9393) in T-PBS/0.2% BSA at 4 °C for 48 h. After subsequent washing 3 × with PBS, slices were incubated with Alexa488 conjugated anti-rabbit secondary antibody for 1 h and counterstained with DAPI. Slices were washed and mounted in mowiol. Some slices were additionally double-stained with primary antibodies against α-smooth muscle actin (1:1000, Novus Biologicals, NB300-978) followed by Alexa546 conjugated anti-goat secondary antibody or with tomato lectin Alexa649 (1:50, Vector Laboratories, DL-1178).

### Data analysis and statistics

The vascular density was calculated with the grid method as described previously by us (Hutter-Schmid et al. 2015). Briefly, images were obtained with Leica DMIRB inverse microscope and Openlab Software at 10 × magnification. These images were overlaid with a 6 × 6 grid using Photoshop (Adobe Photoshop Elements 2.0). In the experimental set I, the number of vessels crossing the outlines of the grid was counted. The total number of vessels crossing the superimposed grid correlates with the degree of vessel density (Fig. 1c). For the experimental set II, laminin + cells in the gap between two organotypic slices were counted manually in three fields at 10 × magnification (Fig. 1d). Cells with a clear shape and visible nuclei were taken into account. Statistical analysis was performed by one-way ANOVA with a subsequent Fisher LSD post hoc test when more groups present, and by an unpaired *T* test with equal variance between two groups. Statistical results were considered significant at *p* < 0.05. All values were given as mean ± standard error of the mean (SEM). Sample size (*n*) always signified the number of animals.

## Results

### Experimental set up I: vessel crossings after a cut

**Fig. 2:**
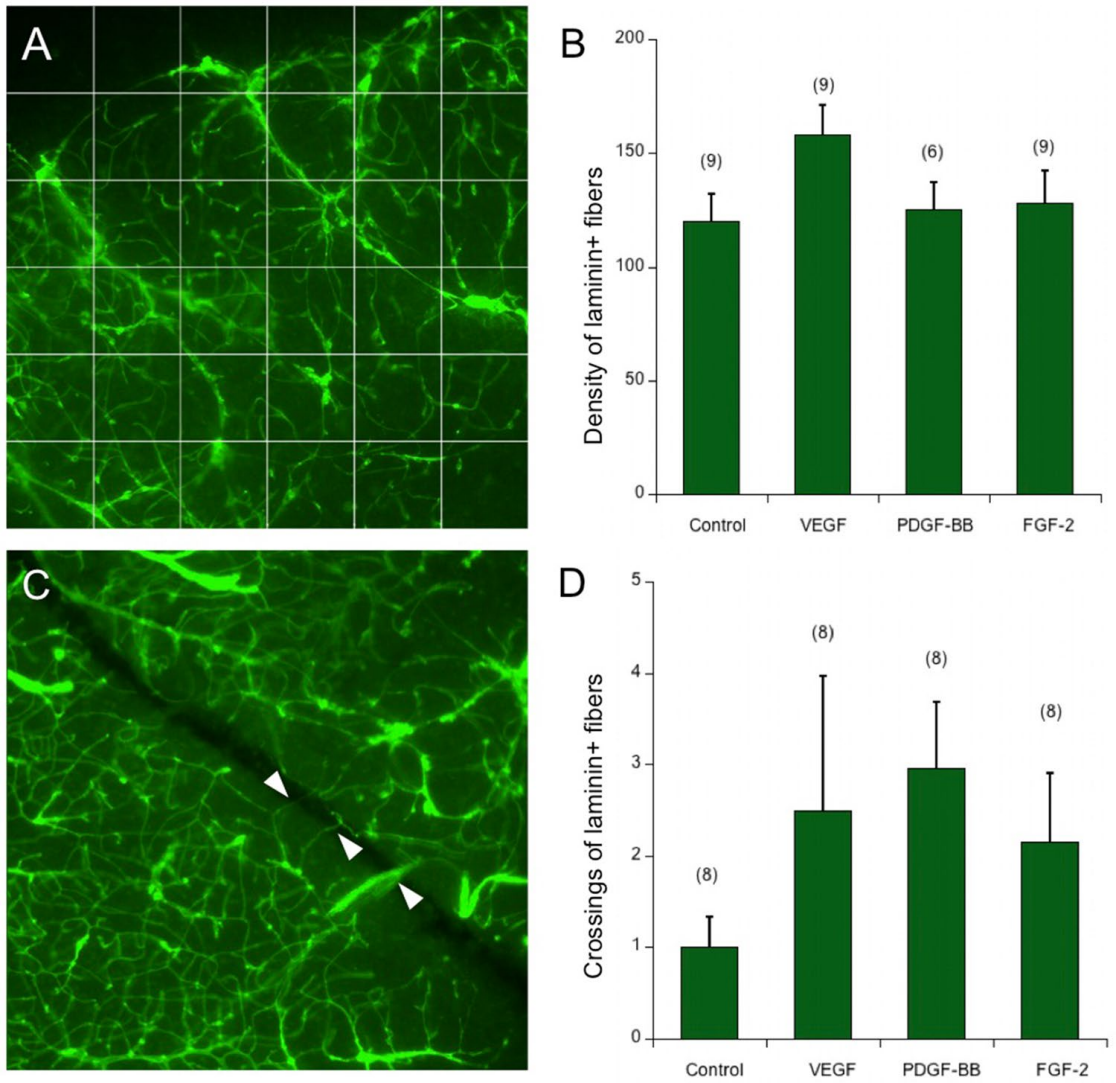
In experimental set I, organotypic brain slices were cultured for 2 weeks either without any supplement (control) or in the presence of 100 ng/ml fibroblast growth factor-2 (FGF-2), vascular endothelial growth factor (VEGF), or platelet-derived growth factor-BB (PDGF-BB) in the media. Slices were stained for laminin and the vessel density was quantified with the help of a superimposed 6 × 6 grid on the microscope images (**a**, **b**). A cut with a scapel was made on the right side of the slices directly after setting up the cultures and the number of vessels crossing this cut (white arrows in **c**) was quantified (**c**, **d**). The image (**c**) was obtained from a slice incubated in PDGF-BB. Statistical analysis was performed by one-way ANOVA with a subsequent Fisher LSD post hoc test. Values in parentheses indicate the number of animals. Note that the laminin vessel density did not change significantly, but there was a tendency for increased vessel crossings in the cut. Scale bar in (**c**): 75 µm (**a**, **c**)

Organotypic brain slices incubated for 2 weeks attached and flattened on the membrane insert (Fig. 1a) and cresyl violet staining showed homogeneous cell layers (Fig. 1b). A scapel cut was made on the right side of the slices (Experimental set up I) and vessel crossings were evaluated in slices incubated with only medium or medium supplemented with 100 ng/ml FGF-2, VEGF, or PDGF-BB. In a control field on the cortex, the vessel density was quantified for the angiogenic factors (Fig. 1c). Vessels that cross a 6 × 6 grid were counted to evaluate the vessel density at the cortex (Fig. 2a). In the control slices incubated with medium without any supplements, 120 ± 12 vessels that cross the 6 × 6 grid were counted (Fig. 2b). Slices incubated with 100 ng/ml angiogenic factors did not exhibit a significant increase in the vessel density in the cortex (Fig. 2b). The addition of VEGF to the medium provided a tendency for an increase in the number of vessels crossing the grid (158 ± 13) compared to the control group, while PDFG-BB (125 ± 12) and FGF-2 (128 ± 14) did not provide a noticeable change. To study spontaneous vessel growth in organotypic brain slices, vessels in the slice were cut with a scapel after the transfer of the slices onto the semipermeable membrane (Fig. 2c). The number of laminin + vessels crossing this cut was 1 ± 0.3 in the control group. In contrast, when slices were incubated with 100 ng/ml VEGF, PDGF-BB, and FGF-2, the number of vessels crossing the cut was increased two-fold (2.4 ± 1.5 for VEGF, 3.0 ± 0.7 for PDGF-BB, and 2.1 ± 0.8 for FGF-2), although not statistically significant (Fig. 2d).

### Experimental set up II: bridging a gap with a hydrogel

**Fig. 3:**
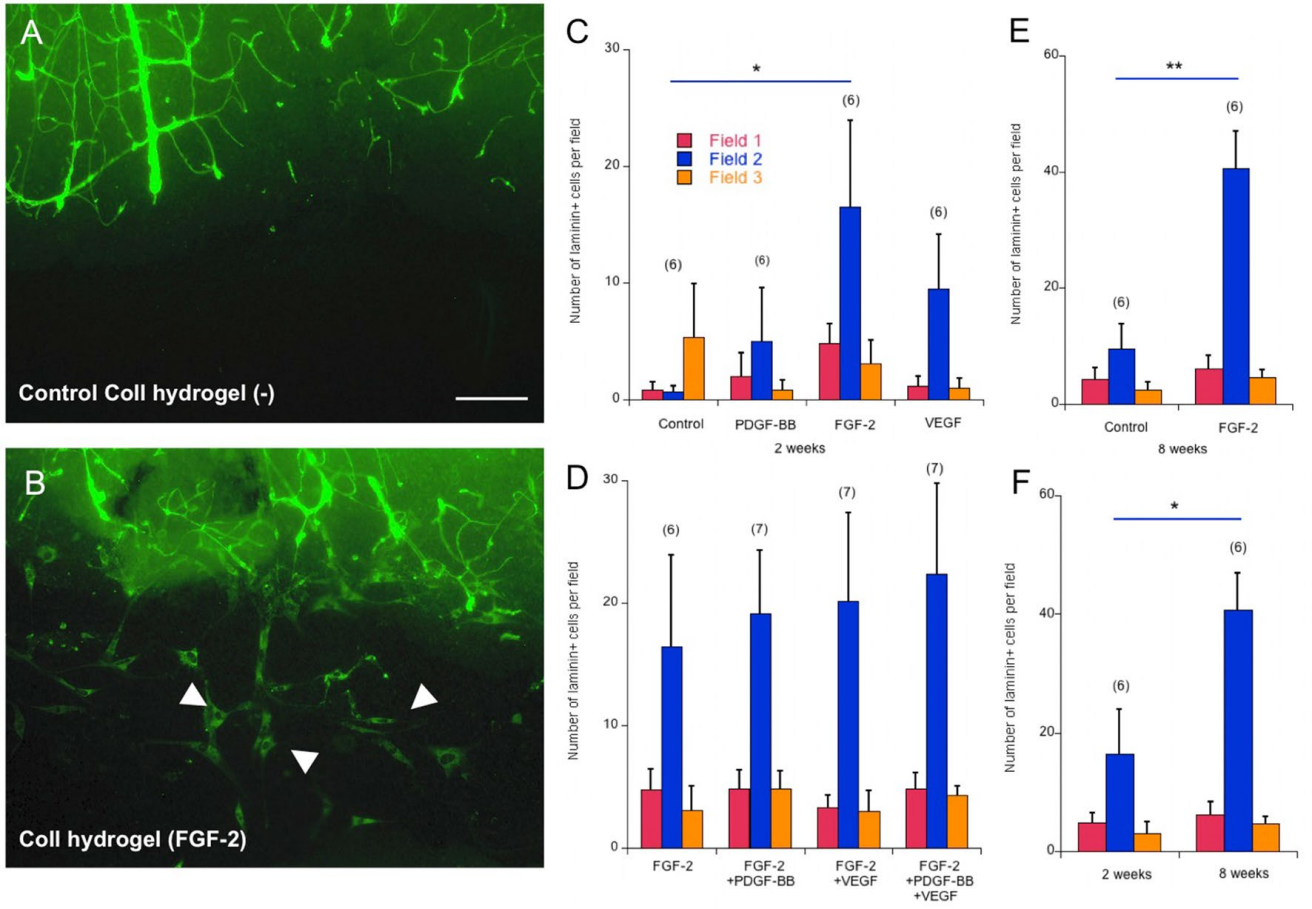
In experimental set II, two coronal half brain slices were incubated with a distance of 1.5 mm for 2 weeks and a hydrogel loaded with or without fibroblast growth factor-2 (FGF-2), vascular endothelial growth factor (VEGF), or platelet-derived growth factor-BB (PDGF-BB) was placed in the gap between the slices. At the end of 2 week culturing, the slices were stained for laminin. Note the laminin + cells (white arrows in **b**) in the gap between the slices with a collagen hydrogel loaded with FGF-2 (**b**) compared to a control hydrogel (**a**). The number of laminin + cells in the gap between slices incubated for 2 weeks was quantified in three fields per each slice pair, one in the location of the collagen hydrogel and two at each side of the slices (**c**). The same quantification was conducted for a combination of FGF-2 with either PDGF-BB, VEGF, or both loaded into collagen hydrogels (**d**). Additionally, the number of laminin + cells for slices incubated for 8 weeks with an empty (control) or FGF-2-loaded collagen hydrogel was evaluated (**e**). FGF-2-loaded collagen hydrogels provided a time-dependent increase in the number of laminin + cells in the location of the hydrogel from 2 to 8 weeks (**f**). Values are given as mean ± SEM. Statistical analysis was performed by one-way ANOVA with a subsequent Fisher LSD post hoc test for (**c**) or an unpaired *T* test with equal variance for (**d**, **e**). (*p < 0.05; **p < 0.01) Values in parentheses indicate the number of animals. Scale bar in (**a**): 75 µm (**a**, **b**)

To study vessel growth, two coronal half brain slices with a distance of 1.4 ± 0.1 mm (*n* = 10) were prepared and a collagen hydrogel was placed in between the next day (Fig. 1d). After incubation of slices for 2 weeks with a collagen hydrogel, laminin + cells were observed in the gap between slices. This effect was most pronounced when the hydrogels were loaded with FGF-2 (Fig. 3a, b). To quantify this effect, the number of laminin + cells was counted in three regions in the gap between the slices, one in the location of a collagen hydrogel (Field 2) and one on each side of the slices (field 1 and 3) (Fig. 3c). The number of laminin + cells between slices was low in the control group in (1 ± 1 cells). Placing an FGF-2-loaded hydrogel resulted in a significant increase in the number of laminin + cells in field 2 (17 ± 7 cells), where the hydrogel was located (Fig. 3c). VEGF and PDGF-BB-loaded hydrogels did not cause any significant increase in the number of laminin + cells at field 2 (10 ± 5 and 5 ± 4 cells, respectively) (Fig. 3c). Therefore, further experiments were carried out with FGF-2-loaded collagen hydrogels.

In this model, we additionally investigated a possible synergistic effect of FGF-2 in combination with PDGF-BB, VEGF, or both. After 2 weeks of incubation, none of these slices had the formation of tube-like structures or de novo vessels. The number of laminin + cells in the location of collagen hydrogel (field 2) was increased compared to collagen hydrogels loaded with FGF-2 alone (Fig. 3d), which was 19 ± 5 for FGF-2 + PDGF-BB and 20 ± 7 for FGF-2 + VEGF, neither statistically significant. A combination of all three angiogenic factors provided the highest number of laminin + cells at field 2 (22 ± 7); however, this was also not significantly higher than the combination of both factors.

Furthermore, two coronal half slices were incubated with a collagen hydrogel that was empty or loaded with FGF-2 for 8 weeks. The number of laminin + cells in between was significantly higher in the field where the FGF-2-loaded collagen hydrogel was placed compared to the control group (Fig. 3e). The number of laminin + cells also increased in a time-dependent manner. This increase was not statistically significant for the control group between 2 and 8 weeks (10 ± 4 cells). The number of cells in the field where FGF-2-loaded collagen hydrogels were placed (field 2) was significantly higher for slices incubated for 8 weeks (41 ± 6 cells) compared to the slices incubated for 2 weeks (Fig. 3f).

For the control fields outside of the region where collagen hydrogels were located (field 1 and 3), there was no significant change for any of the groups or time points. At week 2, these values were for control group 1 ± 1 and 5 ± 5, for PDGF-BB 2 ± 2 and 1 ± 1, for FGF-2 5 ± 1 and 3 ± 2, and for VEGF 1 ± 1 and 1 ± 1 (field 1 and field 3, respectively). In the combination experiment, these values were for FGF-2 + PDGF-BB 5 ± 2 and 5 ± 2, for FGF-2 + VEGF 3 ± 1 and 3 ± 2, for FGF-2 + VEGF + PDGF-BB 5 ± 1 and 4 ± 1 (field 1 and field 3, respectively). At week 8, there was an increase compared to week 2 in control group in the areas outside of empty collagen hydrogels, 4 ± 2 and 3 ± 1 (field 1 and field 3, respectively), which was not significant. Additionally, there was a slight increase for FGF-2 group from week 2 to week 8 in these areas, 6 ± 2 and 5 ± 1 (field 1 and field 3, respectively), which was not significant.

**Fig. 4:**
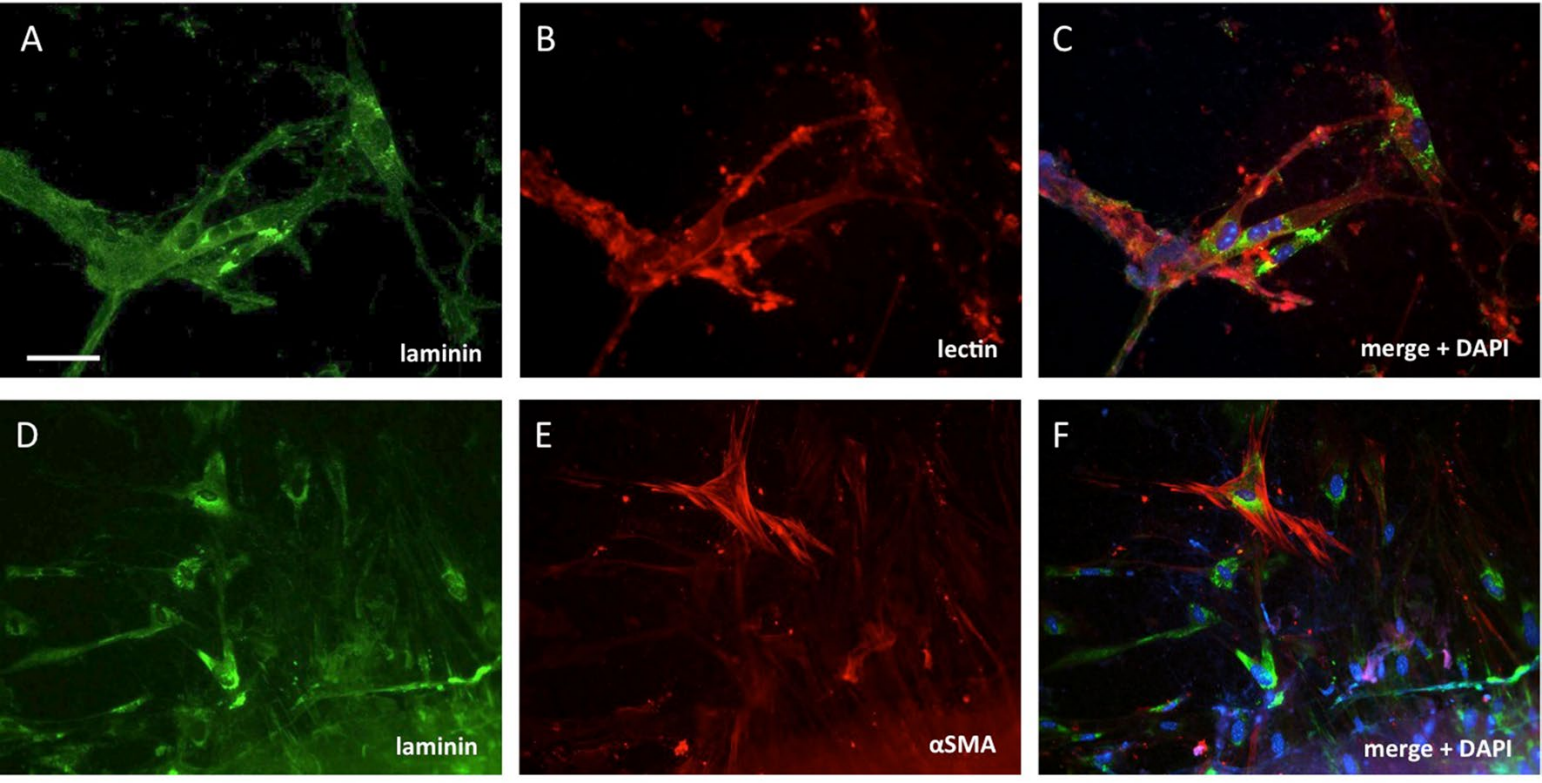
Characterization of migrated cells that were in the gap between the slices stained for laminin (**a** and **d**), lectin (**b**), or alpha-smooth muscle actin (αSMA) (**e**). Slices were incubated for 8 weeks loaded with a fibroblast growth factor-2 (FGF-2) collagen hydrogel. Staining for lectin (**b**) showed co-localization with laminin + migrated cells (**c**), showing long-thin cell bodies with protruding extensions. A few laminin + cells also co-localized with αSMA (**f**). **c** and **f** Merged pictures, where cell nuclei were co-stained with DAPI (blue). Scale bar in (**a**): 25 µm (**a**–**c**) and 50 µm (**d**–**f**)

To characterize the cells in between the slices, co-localization immunostainings were performed for laminin with lectin and a αSMA. Laminin + cells co-localized with lectin for brain vessels (data not shown) and also with migrated cells in the gap between the slices (Fig. 4a–c). These cells displayed an elongated appearance with thin cell bodies and filopodia-like extensions. Some of these cells also co-stained for αSMA (Fig. 4d–f).

## Discussion

In the present study, we show that collagen hydrogels loaded with FGF-2 may provide a “bridging” to enhance vessel re-growth in organotypic brain slices.

### Laminin + vessels in organotypic brain slices

In our research group, we have well documented that in organotypic brain slices, capillaries survive well in the absence of blood flow during culturing up to 4 weeks (Moser et al. 2003). Some components of the brain vasculature degenerate and break down due to the dissection and slicing procedures, as well as the absence of blood flow during culturing (Bendfeldt et al. 2007). Nonetheless, an intact and extensive capillary network was visualized with laminin staining in all slices after 2 weeks or 8 weeks in culture. Laminin is a well-established basement membrane marker used for staining the vessel walls and laminin producing vascular endothelial cells. Therefore, staining for laminin has been successfully used in organotypic brain slices for the examination of vessels (Bendfeldt et al. 2007; Hutter-Schmid et al. 2015). In fact, we show a very dense laminin + network all over the brain slices, but focused on cortical areas in the further experiments.

### Effects of angiogenic factors (experimental set up I)

FGF-2 added to the culture media (100 ng/ml) did not provide a significant improvement in the vessel density at the end of the 2 week culturing period compared to the control group. In a previous study, FGF-2 in low doses was shown to significantly prevent the loss of vessels in cortical organotypic brain slices, while higher doses (50 ng/ml) were ineffective (Bendfeldt et al. 2007). The addition of VEGF (100 ng/ml) resulted in an increase in the density of laminin + vessels, although statistically not significant. VEGF is an important factor also for the maintenance and stabilization of the vessel structures. Our results can point out a protective effect of VEGF on the vessels in organotypic brain slices during culturing. On the other hand, PDGF-BB failed to show any improvement in the vessel density compared to the control group. Indeed, PDGF-BB is involved in smooth muscle and pericyte incorporation onto the vessels, rather than angiogenesis on its own (D’Amore and Smith 1993). When a scapel cut was applied to the slices, spontaneous repair of the blood vessels over the cut was rare. This repair also could not be achieved in significant level by the addition of angiogenic factors, although provided an improvement. These results indicate that there must be additional requirements to be met other than exogenous growth factor support to accomplish vessel repair.

### Collagen hydrogels for release of angiogenic factors

Collagen hydrogels are biocompatible, cause no cytotoxicity, and can be directly applied onto the organotypic brain slices (Ucar and Humpel 2018). Collagen hydrogels crosslinked with PEG release loaded molecules in a degradation-based fashion, in which the loaded molecules are discharged as the surrounding hydrogel is naturally degraded over time (Foidl et al. 2018). Collagen hydrogels are stable in medium or on organotypic brain slices, and degraded within 14 days in culture, as shown in our previous studies (Foidl et al. 2018). Various collagen hydrogel formulations were frequently used as three-dimensional growth matrices to study angiogenesis by embedding endothelial cells or vessel fragments (Hoying et al. 1996; Satake et al. 1998; Koh et al. 2008) or by growing cells on top (Montesano et al. 1986; Bayless et al. 2009). Collagen gels together with FGF-2 treatment provided higher survival of embedded endothelial cells and enhanced vessel branching from embedded vessel fragments (Satake et al. 1998; Nicosia et al. 2005). Collagen hydrogels loaded with FGF-2 were used before incorporated into collagen sponges in periodontal wound healing and provided early ingrowth of vessel-like structures besides promoting healing in different tissue types (Momose et al. 2016). In the light of these studies, collagen hydrogels can be a useful tool for providing an extracellular matrix for endothelial cell proliferation and migration, as well as a modifiable platform for delivering angiogenic factors.

### Effect of angiogenic factors loaded into collagen hydrogels (experimental set up II)

In the next step, we aimed to use collagen hydrogels to bridge two brain slices. We evaluated three areas between the slices. Under normal circumstances, the turnover of vascular endothelial cells is very slow (Folkman 1992). Indeed, a very low number of laminin + cells was seen in the gap between slices with a control hydrogel in between in the slices incubated for 2 weeks. When a hydrogel loaded with FGF-2 was placed between the slices, there were significantly more laminin + cells observed in the area between the slices where the collagen hydrogel was placed (field 2). The laminin + endothelial cells in this gap displayed mostly long, thin cell bodies with extending filopodia-like cellular projections from one side, which are known for guiding cell migration (de Smet et al. 2009). In the fields outside of the hydrogel (fields 1 and 3), there was a very low number of laminin + cells, which was not significantly different from the control group. No statistically significant increases were found in the number of laminin + cells when the hydrogels in between the slices were loaded with VEGF or PDGF-BB. Based on these results and previous data, we focused on FGF-2 in further experiments.

The synergistic effects of FGF-2 and VEGF or PDGF-BB are demonstrated in various models previously. FGF-2 and VEGF in combination had significantly stronger effects on endothelial cell migration and tube-like structure formation in vitro compared to either factors alone (Pepper et al. 1992, 1998). This synergism was later confirmed in an in vivo model, where the combination of FGF-2 and VEGF provided a more potent effect than the effects of FGF-2 and VEGF alone on angiogenic responses such as capillary density (Asahara et al. 1995). In an in vivo model, PDGF-BB and FGF-2 also provided higher vascularization than their individual effects added together, while in this study, the combination of FGF-2 and VEGF did not induce a significant effect (Cao et al. 2003). Therefore, we investigated the effect of the combination of these angiogenic factors with FGF-2 loaded into collagen hydrogels. Slices with collagen hydrogels loaded with a combination of FGF-2 with VEGF, PDGF, or both did not show de novo vessel formation. However, they caused an increase in the number of laminin + cells compared to FGF-2 alone, although this increase was not statistically significant for any of the groups. The largest increase was in the group with collagen hydrogels loaded with all three angiogenic factors.

To follow up long-term effects, slices were incubated for 8 weeks with a collagen hydrogel loaded with FGF-2. In these slices, FGF-2-loaded collagen hydrogels again showed a significant increase in the number of laminin + cells compared to the control hydrogels. Furthermore, the number of laminin + cells for the FGF-2 group was significantly higher when slices were incubated for 8 weeks compared to the slices incubated for 2 weeks. This result indicates that an initial FGF-2 support and an extracellular collagen matrix induce longer term benefits for endothelial cell proliferation and migration, even after the hydrogel is degraded. To characterize the migrated cells, a co-staining was performed for laminin, lectin, and αSMA. Lectin co-localized well with laminin not only for the vessels in the slices, but also for the migrated endothelial cells in the gap between the slices. In the brain vasculature αSMA is a marker for pericytes and vascular smooth muscle cells (Smyth et al. 2018). Staining for αSMA showed a few laminin /lectin + cells that were surrounded by αSMA + fiber bundles. Thus, our data indicate that, indeed, FGF-2 promotes the migration of endothelial cells, but not new vessel formation.

### Limitations and future perspectives

This study provides proof of concept that collagen hydrogels loaded with FGF-2 can be used as an extracellular matrix for proliferation and migration of vascular endothelial cells. Collagen hydrogels have the potential to provide a flexible platform as an external matrix with considerable possibilities for modification of mechanical properties, degradation rate, and incorporated substances. Formation of new capillaries depends on proliferation of endothelial cells, local degradation of basement membrane, and endothelial migration. FGF-2 takes part in all these processes. However, a cocktail of growth factors and angiogenesis modulators seems to be necessary to induce vessel formation. These factors could also be applied in collagen hydrogels. The slice bridging technique can be used to study different neurodegenerative conditions, but also peripheral nerve injuries or spinal cord lesions, as well as local vascular damage. More experiments are necessary in vivo to demonstrate the potential of FGF-2-loaded collagen hydrogels to induce vessel formation.

## Conclusion

In conclusion, our data provide evidence that FGF-2 induced proliferation and migration of laminin + endothelial cells into the gap between two brain slices. None of these cells formed tube-like structures, but instead spread as a monolayer. Collagen hydrogels loaded with a mixture of different angiogenic growth factors may provide a potent tool in brain vessel repair.

## References

[CR1] Alberts B, Johnson A, Lewis J, Raff M, Roberts K, Walter P (2002). Molecular biology of the cell.

[CR2] Asahara T, Bauters C, Zheng LP, Takeshita S, Bunting S, Ferrara N, Symes JF, Isner JM (1995). Synergistic effect of vascular endothelial growth factor and basic fibroblast growth factor on angiogenesis in vivo. Circulation.

[CR3] Battegay EJ, Rupp J, Iruela-Arispe L, Sage EH, Pech M (1994). PDGF-BB modulates endothelial proliferation and angiogenesis in vitro via PDGF beta-receptors. J Cell Biol.

[CR4] Bayless KJ, Kwak HI, Su SC (2009). Investigating endothelial invasion and sprouting behavior in three-dimensional collagen matrices. Nat Protoc.

[CR5] Bellacen K, Lewis EC (2009). Aortic ring assay. JOVI.

[CR6] Bendfeldt K, Radojevic V, Kapfhammer J, Nitsch C (2007). Basic fibroblast growth factor modulates density of blood vessels and preserves tight junctions in organotypic cortical cultures of mice: a new in vitro model of the blood-brain barrier. J Neurosci.

[CR7] Cao R, Brakenhielm E, Pawliuk R, Wariaro D, Post MJ, Wahlberg E, Leboulch P, Cao Y (2003). Angiogenic synergism, vascular stability and improvement of hind-limb ischemia by a combination of PDGF-BB and FGF-2. Nat Med.

[CR8] Cao R, Eriksson A, Kubo H, Alitalo K, Cao Y, Thyberg J (2004). Comparative evaluation of FGF-2-, VEGF-A-, and VEGF-C-induced angiogenesis, lymphangiogenesis, vascular fenestrations, and permeability. Circ Res.

[CR9] Cipolla MJ (2009). The cerebral circulation. Chapter 2, anatomy and ultrastructure.

[CR10] D'amore P, Smith SR (1993). Growth factor effects on cells of the vascular wall: a survey. Growth Factors.

[CR11] De Smet F, Segura I, De Bock K, Hohensinner PJ, Carmeliet P (2009). Mechanisms of vessel branching: filopodia on endothelial tip cells lead the way. Arterioscler Thromb Vasc Biol.

[CR12] Ferrara N, Gerber HP, LeCouter J (2003). The biology of VEGF and its receptors. Nat Med.

[CR13] Foidl BM, Ucar B, Schwarza A, Rebelob AL, Pandit A, Humpel C (2018). Nerve growth factor released from collagen scaffolds protects axotomized cholinergic neurons of the basal nucleus of Meynert in organotypic brain slices. J Neurosci Methods.

[CR14] Folkman C (1992). Angiogenesis. J Biol Chem.

[CR15] Hoying JB, Boswell CA, Williams SK (1996). Angiogenic potential of microvessel fragments established in three-dimensional collagen gels. Vitro Cell Dev Biol Anim.

[CR16] Hu X, De Silva TM, Chen J, Faraci FM (2017). Cerebral vascular disease and neurovascular injury in ischemic stroke. Circ Res.

[CR17] Hughes CS, Postovit LM, Lajoie GA (2010). Matrigel: a complex protein mixture required for optimal growth of cell culture. Proteomics.

[CR18] Humpel C (2015). Organotypic brain slice cultures: a review. Neuroscience.

[CR19] Hutter-Schmid B, Kniewallner KM, Humpel C (2015). Organotypic brain slice cultures as a model to study angiogenesis of brain vessels. Front Cell Dev Biol.

[CR20] Kapoor A, Chen CG, Iozzo RV (2020). A simplified aortic ring assay: a useful ex vivo method to assess biochemical and functional parameters of angiogenesis. Matrix Biol Plus.

[CR21] Khurana R, Simons M (2003). Insights from angiogenesis trials using fibroblast growth factor for advanced arteriosclerotic disease. Trends Cardiovasc Med.

[CR22] Koh W, Sachidanandam K, Sacharidou A, Davis GE (2008). In vitro three dimensional collagen matrix models of endothelial lumen formation during vasculogenesis and angiogenesis. Methods Enzymol.

[CR23] Momose T, Miyaji H, Kato A, Ogawa K, Yoshida T, Nishida E, Murakami S, Kosen Y, Sugaya T, Kawanami M (2016). Collagen hydrogel scaffold and fibroblast growth factor-2 accelerate periodontal healing of class II furcation defects in dog. Open Dent J.

[CR24] Montesano R, Vassalli JD, Baird A, Guillemin R, Orci L (1986). Basic fibroblast growth factor induces angiogenesis in vitro. Proc Natl Acad Sci USA.

[CR25] Moser KV, Schmidt-Kastner R, Hinterhuber H, Humpel C (2003). Brain capillaries and cholinergic neurons persist in organotypic brain slices in the absence of blood flow. Eur J Neurosci.

[CR26] Nicosia RF, Zhu WH, Fogel E, Howson KM, Aplin AC (2005). A new ex vivo model to study venous angiogenesis and arterio-venous anastomosis formation. J Vasc Res.

[CR27] Oudega M (2012). Molecular and cellular mechanisms underlying the role of blood vessels in spinal cord injury and repair. Cell Tissue Res.

[CR28] Park CM, Hollenberg MJ (1989). Basic fibroblast growth factor induces retinal regeneration in vivo. Dev Biol.

[CR29] Pepper MS, Ferrara N, Orci L, Montesano R (1992). Potent synergism between vascular endothelial growth factor and basic fibroblast growth factor in the induction of angiogenesis in vitro. Biochem Biophys Res Commun.

[CR30] Pepper MS, Mandriota SJ, Jeltsch M, Kumar V, Alitalo K (1998). Vascular endothelial growth factor (VEGF)-C synergizes with basic fibroblast growth factor and VEGF in the induction of angiogenesis in vitro and alters endothelial cell extracellular proteolytic activity. J Cell Physiol.

[CR31] Presta M, Dell'Era P, Mitola S, Moroni E, Ronca R, Rusnati M (2005). Fibroblast growth factor/fibroblast growth factor receptor system in angiogenesis. Cytokine Growth Factor Rev.

[CR32] Satake S, Kuzuya M, Ramos MA, Kanda S, Iguchi A (1998). Angiogenic stimuli are essential for survival of vascular endothelial cells in three-dimensional collagen lattice. Biochem Biophys Res Commun.

[CR33] Slavin J (1995). Fibroblast growth factors: at the heart of angiogenesis. Cell Biol Int.

[CR34] Smyth LCD, Rustenhoven J, Scotter EL, Schweder P, Faull RLM, Park TIH, Dragunow M (2018). Markers for human brain pericytes and smooth muscle cells. J Chem Neuroanat.

[CR35] Ucar B, Humpel C (2018). Collagen for brain repair: therapeutic perspectives. Neural Regen Res.

[CR36] Ucar B, Humpel C (2019). Therapeutic efficacy of glial cell-derived neurotrophic factor loaded collagen scaffolds in ex vivo organotypic brain slice Parkinson’s disease models. Brain Res Bull.

[CR37] Vukicevic S, Kleinman HK, Luyten FP, Roberts AB, Roche NS, Reddi AH (1992). Identification of multiple active growth factors in basement membrane Matrigel suggests caution in interpretation of cellular activity related to extracellular matrix components. Exp Cell Res.

[CR38] Zlokovic BV (2005). Neurovascular mechanisms of Alzheimer’s neurodegeneration. Trends Neurosci.

